# An Efficient Printing Defect Detection Based on YOLOv5-DCN-LSK

**DOI:** 10.3390/s24237429

**Published:** 2024-11-21

**Authors:** Jie Liu, Zelong Cai, Kuanfang He, Chengqiang Huang, Xianxin Lin, Zhenyong Liu, Zhicong Li, Minsheng Chen

**Affiliations:** School of Mechatronics Engineering and Automation, Foshan University, Foshan 528225, China

**Keywords:** printing defect detection, YOLOv5, deep learning

## Abstract

During the production process of inkjet printing labels, printing defects can occur, affecting the readability of product information. The distinctive shapes and subtlety of printing defects present a significant challenge for achieving high accuracy and rapid detection in existing deep learning-based defect detection systems. To overcome this problem, we propose an improved model based on the structure of the YOLOv5 network to enhance the detection performance of printing defects. The main improvements include the following: First, we introduce the C3-DCN module to replace the C3 module in the backbone network, enhancing the model’s ability to detect narrow and elongated defects. Secondly, we incorporate the Large Selective Kernel (LSK) and RepConv modules into the feature fusion network, while also integrating a loss function that combines Normalized Gaussian Wasserstein Distance (NWD) with Efficient IoU (EIoU) to enhance the model’s focus on small targets. Finally, we apply model pruning techniques to reduce the model’s size and parameter count, thereby achieving faster detection. Experimental results demonstrate that the improved YOLOv5 achieved a mAP@0.5 of 0.741 after training, with 323.2 FPS, which is 2.7 and 20.8% higher than that of YOLOv5, respectively. The method meets the requirements of high precision and high efficiency for printing defect detection.

## 1. Introduction

**Figure 1:**
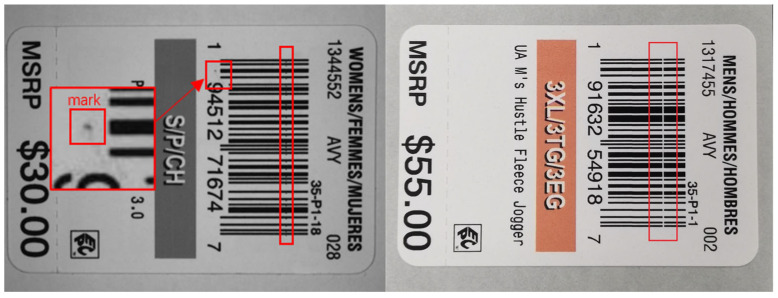
Sample of label printing defect.

In recent years, automatic detection of inkjet printing defects has become a crucial aspect in ensuring the quality and reliability of the final printed product. This topic has received significant attention in the field. During the printing production process, numerous factors, including machine vibration, the production environment, and others, can result in the occurrence of defects, such as white lines, stains, etc., as shown in Figure 1. The primary objective of defect detection is not merely to ascertain the presence or absence of defects but also to obtain data on the number, area, and types of defects, as well as their positions relative to the critical printed content. These factors are of paramount importance in determining the conformity of a printed product. For instance, specific minor imperfections may be tolerable under certain circumstances, whereas more substantial defects could potentially result in product failure. Defects occurring on crucial information such as Quick Response (QR) codes and the product’s logo, even if they are minor, have the potential to impact the brand image and readability of the merchandise. On the other hand, the type of defect is frequently indicative of the underlying cause, which in turn provides insight into the malfunction of the hardware system. For instance, mark defects may be attributed to a number of factors, including fouling of the inkjet head, deposition of ink dots, contamination of the printhead, which causes impurities to be ejected alongside the ink, or quality issues with the ink itself. Consequently, precise defect detection can provide a scientific foundation for quality control, assist the production line in making informed decisions, minimize the financial loss associated with substandard products, and enhance economic efficiency [1]. 

In the early stages of the printing inspection industry, inkjet printing defect detection methods were primarily manual visual inspection and instrumentation detection. However, the manual visual inspection method is subject to subjectivity, visual fatigue, slow detection speed, and other shortcomings. Instrumentation detection requires the use of specialized instruments and incurs high detection costs, and it is unable to detect multiple defects simultaneously [2]. Currently, digital image algorithm inspection has been applied in numerous industrial production settings [3]. In contrast, digital image algorithm detection is a non-contact detection method based on image processing technology. It involves matching the image to be detected with a template and then comparing the two images to identify any differences [4]. However, the application of this method is prone to misjudgment in the detection of print defects with complex print backgrounds, variable contents, and random sizes of defect morphology, and requires the design of detection algorithms according to different defects with poor generalization ability. Therefore, the use of a deep learning target detection algorithm becomes a more effective solution.

In recent years, deep learning methods have been widely used in the field of target detection, including defect detection [5], medical image analysis [6], and security monitoring [7]. Target detection algorithms can be divided into two-stage and one-stage. The two-stage network divides the detection problem into two stages: firstly, generating candidate regions, and then classifying the candidate regions after position refinement. Its main representative algorithms are SPP-net [8] and Faster RCNN [9]. One-stage network detection directly generates the probability of the object category and the object position coordinate values, which has a faster detection speed and better meets the demands of real-time detection scenarios compared with a two-stage network. Its main representative algorithms are SSD [10] and YOLO [11]. In recent years, there have been several studies exploring the application of deep learning models in printing defect detection. Li, Jing et al. [12] employed ResNet as a feature extraction network, weighting the loss function according to the number of samples to enhance alignment accuracy and recognition efficiency. Liu, Andong et al. [13] proposed a double sparse low-rank decomposition method, which decomposes based on a print prior and defects to improve the detection of complex irregular defects. Zhang, Erhu et al. [14] proposed an edge-guided differential attention network to enhance the visibility of defective regions by emphasizing edge information while incorporating top-down attention to reduce the visual clutter caused by background regions. Li, Dongming et al. [15] proposed a patch-based multiscale pyramid registration network to enhance the alignment capability of large distortions while introducing a distortion loss function to improve the text distortion problem.

Despite the progress made in the current method, several challenges remain to be addressed: (1) The current deep learning-based technology faces obstacles in realizing the accuracy of detecting printing subtle defects, including printing defects with their unique narrow and long defects and the difficulty of detecting small defects. (2) The current models for small target detection typically employ models with a large number of parameters, which present two significant challenges: a prolonged inference time and a sizeable model file. This inherent complexity makes it difficult to achieve the desired detection accuracy and speed simultaneously. To address the aforementioned issues, this paper proposes an advanced and efficient detection network model to address the challenges faced by print defect detection. The model is based on the improved YOLOv5 architecture, which can detect various small-scale targets in complex images with greater accuracy and speed. We made improvements to the baseline model in the following aspects:

1. We replaced the C3 module in the backbone network with the C3-DCN module, enabling adaptive refinement of the ROI and flexible adjustment of convolutional kernel shapes. This modification significantly improved the network’s ability to detect elongated defects in printed materials.
2. To improve the detection of minute defects, we integrated the LSK-RepConv module between the neck and prediction layers. Additionally, we proposed the WEIoU loss function, which combined NWD with EIoU to better assess anchor box similarity, thereby enhancing feature extraction for small-scale objects and boosting detection accuracy.
3. To ensure compatibility with low-power mobile devices and achieve a balance between accuracy and speed, we adopted a lightweight design for the improved model. Model pruning is employed to remove redundant weights, reducing model complexity and inference time.

The rest of the paper is organized as follows: Section 2 presents the YOLOv5 improved method, which describes the C3-DCN, LSK-RepConv, and WEIoU loss functions in detail. Section 3 presents the dataset, experimental setup, evaluation metrics, and analysis of the experimental results to quantitatively validate the effectiveness of the proposed method. Finally, Section 4 summarizes the full paper and outlines future works.

## 2. Method

### 2.1. Improved-YOLOv5

In recent years, the YOLO (You Only Look Once) family of models has made significant progress in the field of target detection, attracting widespread attention for its high accuracy and fast inference speed. In order to determine the most suitable solution for print defect detection, we evaluated the performance of YOLO models commonly used in industrial applications in terms of detection accuracy, inference speed, and complex scene adaptability in the same dataset. The results are shown in Table 1.

Although v7 and v8 show slightly higher detection performance compared to the rest of the series, their increased structural complexity could pose challenges on devices with limited processing power. For instance, YOLOv8 incorporates more complex modules into its design, including deeper convolutional layers, advanced attention mechanisms, and enhanced feature fusion modules. While these additions improve detection accuracy, they also substantially increase computational requirements. The GFLOPs of YOLOv8 are approximately twice as large as that of YOLOv5, and thus the inference speed is significantly slower, especially on embedded devices with limited resources. In addition, YOLOv7 presents challenges in portability when converting to TensorRT or ONNX formats, as the associated tools and optimizations remain relatively immature, resulting in suboptimal compatibility and usability. Given our plans to deploy the model on embedded devices, YOLOv5’s relatively simple architecture makes it a more practical choice, as it balances performance with computational efficiency. Moreover, YOLOv5 benefits from extensive optimization, a mature toolchain, and strong support for model conversion and deployment, enabling it to adapt more reliably and efficiently to a variety of hardware platforms. After considering various factors, we finally selected YOLOv5 as the benchmark model for this study.

The YOLOv5 algorithm employs the CIoU loss function for regression of prediction frames, with the prediction results obtained through non-extremely large value suppression, filtering, and combining multiple frames of the network output. The network structure primarily comprises four components: input, backbone network, neck network, and prediction head network. The input is the sample image to be detected. The backbone network is the primary feature extractor of the network, comprising three components: the CBS, C3, and Spatial Pyramid Pooling-Fast (SPPF [16]) module. The CBS module incorporates convolution, batch normalization, and SILU activation functions. The C3 module is a modified version of the CSPNet [17], comprising three standard convolutional layers and a bottleneck module. The SPPF employs serial maximal pooling for feature extraction and fusion of multi-scale feature maps, thereby enhancing the feature representation in the sensory field. The main role of the neck network is to integrate the features extracted by the backbone network, thereby enhancing the detection accuracy. This integration is achieved through two distinct structures: FPN and PAN. The FPN network is a top-down structure based on up-sampling, which facilitates the transfer of high-level semantic information. In contrast, the PAN network is a bottom-up structure based on down-sampling, which enables the transfer of shallow image information. Both of these networks facilitate the fusion of feature information, thereby ensuring the optimal preservation of large- and small-scale target information. The main role of the predictive head network is then to predict the fused feature information generated by the neck network, generating several prediction frames.

**Figure 2:**
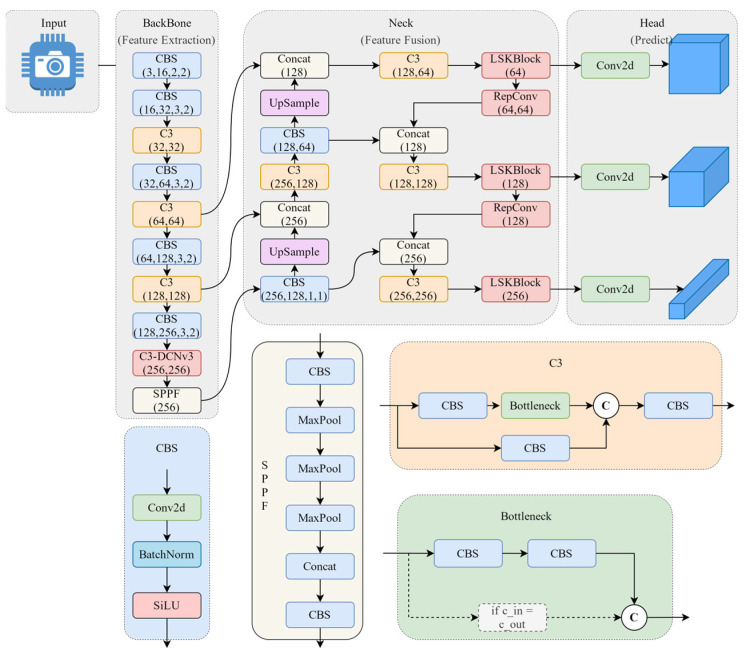
Improved network architecture of YOLOv5.

In this section, we propose an enhanced network architecture based on YOLOv5, as shown in Figure 2. Firstly, we add a DCNv3 module into the C3 module before the SPP layer. This modification enhances the model’s capability to extract and adapt target features of various shapes by adaptively adjusting the shape of the convolutional kernel, hence improving the performance and accuracy of the network. Secondly, we introduce a spatial selection mechanism called LSK-RepConv, which combines LSKBlock with the RepConv module. This mechanism can select feature maps generated by convolutional kernels at different scales to improve the detection of small targets. Thirdly, we propose the WEIoU as a loss function for localization loss. This loss function takes into account both the height and width loss of the prediction frame and the ground truth frame, as well as their similarity. The WEIoU loss function can effectively improve the convergence accuracy of the anchor box, solve the problem of large localization errors of traditional IoU for small targets, and achieve more accurate prediction box localization. Finally, the structured pruning method is applied to reduce the number of parameters while maintaining accuracy.

#### 2.1.1. C3-DCN

**Figure 3:**
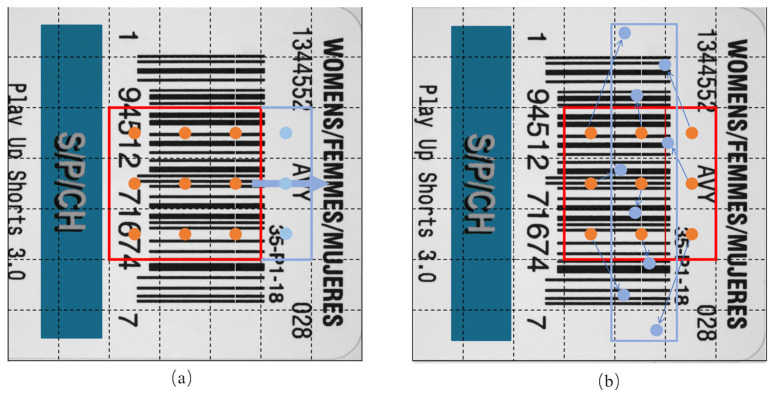
Deformable Convolution Network (DCN) schematic diagram. ((**a**): traditional convolution and (**b**): deformable convolution).

A convolutional neural network extracts the input data by means of a convolution operation and learns the features in the data by continuously adjusting the weight size of the convolution kernel. However, the shape and size of the convolution kernel of CNN are fixed, and its ability to adapt to narrow and long defects, such as white line offset, is limited. In 2017, Dai, Feng et al. [18] proposed deformable convolution, which enhances the spatial sampling locations in the module with additional offsets and learns the offsets from the target task. The defects of narrow and long shapes, which are a distinctive feature of the printing defect detection methodology explored in this paper, present certain limitations when dealing with such shapes using fixed convolutional kernel shapes and sizes, potentially leading to leakage or misdetection. In order to address this challenge, this paper proposes the use of deformable convolutions to adapt the convolutional kernel shape in a manner that better aligns with the characteristics of narrow and long shapes. The comparison of traditional convolution and deformable convolution is shown in Figure 3.

**Figure 4:**
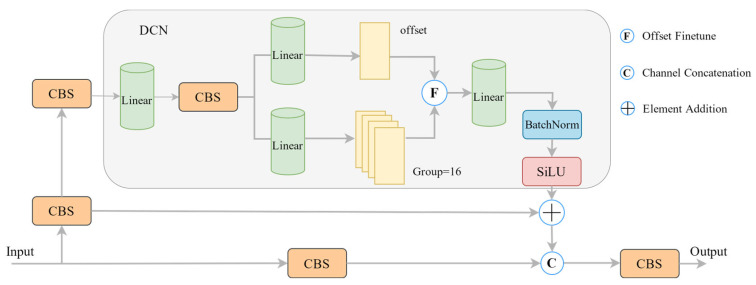
C3 module integrated into DCN [19].

In this paper, we incorporated the DCNv3 [19] module into the C3 module of the backbone network. The DCNv3 module dynamically adjusts the shape of the convolution kernel according to the shape and location of the target, thereby enhancing the network’s ability to identify challenging data, such as label printing defects. The DCNv3 module also introduces a multi-group mechanism and weight coefficients based on the v2, which can be expressed as follows:

(1) $$y\left(p_{0}\right)=\sum_{g=1}^{G}\sum_{k=1}^{k}w_{g}m_{gk}x_{g}\left(p_{0}+p_{k}+∆p_{gk}\right)$$

where *G* is the total number of spatial aggregation groups. For group *g*, $w_{g}$ is the weight of the *k*^th^ sample point, $∆p_{gk}$ is the learnable offset of the *k*^th^ sample point, $p_{k}$ is a 3 × 3 convolution matrix, and $m_{gk}$ is the modulation scalar of the *k*^th^ sample point. SoftMax normalization is used to constrain the range of values between 0 and 1. Additionally, the sum of modulation scalars in the group is constrained to 1 to alleviate the problem of gradient instability. DCNv3 divides the convolutional spatial aggregation process into G groups and calculates the offsets and weights of each sampling point in multiple groups to obtain different spatial aggregation patterns, enabling the network to better extract features. The network structure of C3-DCN is described in Figure 4.

#### 2.1.2. LSK-RepConv

In contrast to conventional target detection, print defects are frequently minute targets with diverse morphologies, rendering their identification more challenging. In order to enhance the efficacy of small target detection in YOLOv5, we have integrated the LSKBlock and RepConv modules into the network architecture before the prediction header. In 2023, Li, Yuxuan et al. [20] proposed a spatial selection mechanism, LSKNet, which aims to enhance the network’s capacity to focus on the most pertinent spatial information while simultaneously enabling the detection of small targets through the spatially selective extraction of feature maps from convolutional kernels of varying scales. 

**Figure 5:**
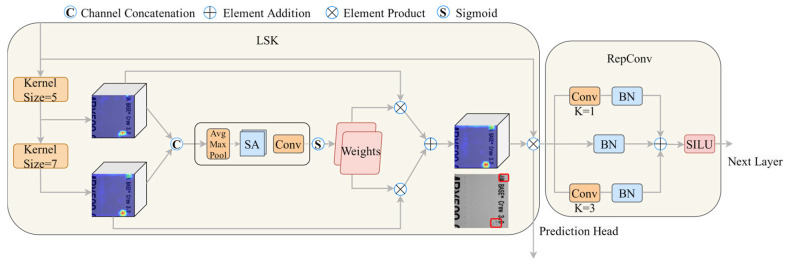
LSK-RepConv module [20].

Figure 5 illustrates the network structure of LSK-RepConv, which merges feature maps $U_{i}$ generated from different convolutional kernels, followed by applying average pooling ${Ρ}_{avg}$ and maximum pooling ${Ρ}_{max}$ to extract the spatial information of the feature maps. The resulting feature maps are then connected and transformed into spatial attention maps using the convolutional operation F.

(2) $$U_{i}=F_{i}\left(X\right)$$

(3) $$\bar{SA_{i}}=F\left(\left[{Ρ}_{max}\left(\sum_{i=1}^{N}U_{i}\right);{Ρ}_{avg}\left(\sum_{i=1}^{N}U_{i}\right)\right]\right)$$

where $F_{i}(\cdot )$ is the convolution operation for different sized convolution kernels, F(·) is the convolution operation, X is the original input feature map for this model, and ${Ρ}_{max}(\cdot )$ and ${Ρ}_{avg}(\cdot )$ are the maximum pooling and average pooling. The sigmoid activation function is used to obtain spatial selection masks for different convolutional kernels from each spatial attention map $\bar{SA_{i}}$. The generated masks are then applied to the corresponding feature maps, which generate spatial attention features through the convolutional layer. Finally, these features are multiplied with the input X to obtain the final output Y.

(4) $$Y=X⨂F(\sum_{i=1}^{N}\left(\delta (\bar{SA_{i}}\right)\cdot U_{i}))$$

where δ(·) is the sigmoid activation function and ⨂ is the matrix product. Compared to conventional spatial attention mechanisms, LSKBlock combines convolutional kernels of different scales for spatial selection. Convolutional kernels with larger sizes have higher accuracy in predicting the position of small targets.

Due to the significant computational overhead associated with the complex attention calculations in the LSKBlock, we have incorporated a RepConv module following the LSKBlock to address the inference speed requirements in practical applications. RepConv, through its unique structural reparameterization design, efficiently processes the spatially enhanced features extracted by the LSKBlock. It maintains feature discriminability while significantly improving inference efficiency. By utilizing structural reparameterization, RepConv retains the expressive capability of a multi-branch structure during training and converts it into an efficient single-branch structure for inference. This sequential module design achieves an ideal balance between detection accuracy and computational efficiency, ensuring that the model’s inference time meets practical production requirements.

#### 2.1.3. WEIoU

The commonly used loss functions, such as CIoU, GIoU, and DIoU, typically focus on single aspects like the distance between the minimum enclosing boxes (GIoU), the distance between bounding box centers (DIoU), or the aspect ratio of bounding boxes (CIoU). While these functions perform well in most object detection tasks, they encounter limitations in detecting subtle and elongated defects in printing applications. Specifically, in cases where anchor boxes do not overlap, these functions often result in significant location bias and cannot accurately measure similarity, which can lead to issues such as gradient explosion or gradient vanishing. Therefore, we adopt the WEIoU loss function to ensure more accurate anchor box predictions.

WEIOU is based on EIoU [21] and incorporates NWD [22] to enhance performance on small targets. EIoU considers three aspects when regressing anchor frames: overlap loss, center distance loss, and height and width loss. Meanwhile, NWD measures the similarity of two probability distributions, with its main advantage being the smoothness of positional deviation. It can effectively measure the similarity of two anchor frame distributions even if they do not overlap or have minimal overlap. NWD considers the entire shape and structure of the two distributions, not just their overlapping parts. The WEIoU loss function combines the multi-factor regression advantages of EIoU with the smooth positional bias measurement benefits of NWD. Even in cases where anchor boxes have minimal or no overlap, WEIoU accurately reflects the precision of anchor box regression, enabling stable optimization of anchor box positioning.

WEIou loss can be formulated as follows:

(5) $$L=\alpha L_{Eiou}+{\left(1-\alpha \right)L}_{NWD}$$

(6) $$L_{Eiou}=1-IOU+\frac{\rho_{Center}^{2}}{C_{Center}^{2}}+\frac{\rho_{Weight}^{2}}{C_{Weight}^{2}}+\frac{\rho_{High}^{2}}{C_{High}^{2}}$$

where $\rho_{\left(\cdot \right)}$ is the distance between the center, width, and height of the two anchor frames, and $C_{\left(\cdot \right)}$ is the diagonal length, height, and width of the two anchor frames’ smallest enclosing box. And $L_{NWD}$ loss can be expressed as follows:

(7) $$\;L_{NWD}=1-\exp\left(-\frac{\sqrt{M_{Center}+M_{wh}}}{Constant}\right)\;=1-\exp\left(-\frac{\sqrt{\Delta x^{2}+\Delta y^{2}+\frac{\Delta w^{2}+\Delta h^{2}}{4}}}{Constant}\right)$$

where Δx and Δy are the positioning differences between the center points of the two anchor frames, Δw and Δh are the differences between the width and height of the two anchor frames, Constant is a constant equal to 12.8, and eps = 1 × 10^−7^ is added to all of the above differences to prevent the phenomenon of Loss being 0 when the prediction frames are completely overlapped with the ground truth. Table 2 shows that WEIoU achieves the highest scores for mAP@0.75 and mAP@0.5:0.95, indicating superior localization accuracy compared to other loss functions. This suggests that WEIoU is particularly effective for detecting narrow and small defects, making it well-suited for high-precision tasks in printing defect detection.

#### 2.1.4. Model Compression by Pruning

**Figure 6:**
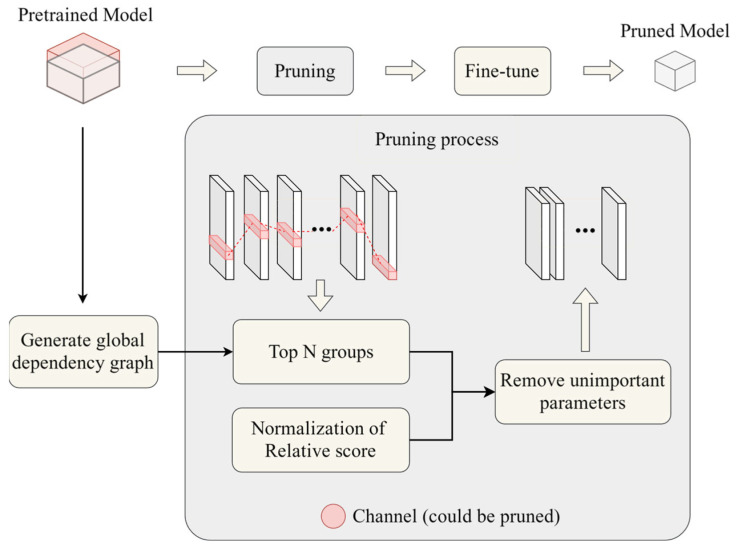
Group Norm pruning method.

Some of the convolutional kernels used to extract image features during the training process may have very low percentage weights in the detection process. The problems of many redundant parameters and poor real-time deployment are often purely in the improved YOLOv5 model. In this section, we prune the improved YOLOv5 model using a structured pruning method called GroupNorm [23]. The method first analyzes the network to construct a dependency graph, which can reflect the dependency relationships among the nodes in the network, and then selects the layers to be pruned and specifies the corresponding pruning channels according to the specific pruning strategies and goals. Finally, according to the grouping information, the specified pruning channels are removed one by one in the order of groups, and the process is shown in Figure 6. This method can reduce the number of parameters in the model and the inference time while having minimal impact on the model’s accuracy. It is suitable for use in embedded devices.

## 3. Experiments and Analyses

This section describes the dataset, experimental setup, experimental evaluation metrics, and experimental results.

### 3.1. Dataset

**Figure 7:**
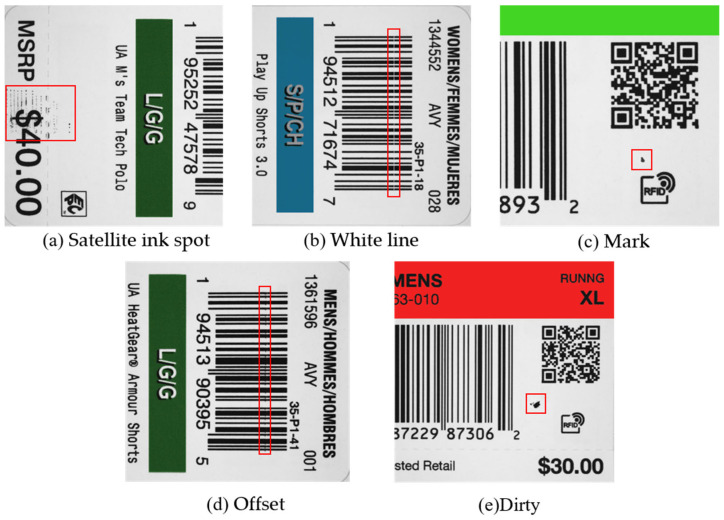
Partial images of the dataset.

We use a dataset provided by a printing company for training and validation. The dataset consists of images of real industrial label printing defects, including five common types of defects: white line, mark, offset, dirty, and satellite ink spot. All of them are printing defects that often occur during the production process due to inkjet nozzle faults, mechanical shuddering, etc. The images were captured by an industrial camera with a resolution of 3400 × 2565; some images from the dataset are shown in Figure 7. The pre-processing of the data includes gray-scaling and resizing to 1376 × 1024. This is done to adapt to the down-sampling module of YOLOv5 and to reduce the number of model parameters, thus speeding up the inference time of the model. In order to avoid overfitting, poor generalization, and detection problems during training, the training set needs to be augmented with data. In order to have an even distribution of labels across the various categories, we finally constructed the dataset: the training set consists of more than 3000 images, the validation set consists of 360 images, and the test set consists of 360 images. The distribution of the various defects in the dataset is shown in Table 3.

### 3.2. Experimental Setup

In this experiment, we used Python 3.8.13 and torch 1.13.1 development environments with NVIDIA RTX3090 GPUs, 24GB RAM, and CUDA11.7. Parameter details for this experiment are shown in Table 4.

### 3.3. Experimental Evaluation Metrics

In the field of target detection, models are commonly evaluated using precision, recall, mAP (mean average precision), parameters, FLOPs (floating point operations), and inference as performance metrics. ‘P’ denotes precision and ‘R’ denotes recall rate, which are calculated as shown in Equations (8) and (9).

(8) $$P=\frac{TP}{TP+FP}$$

(9) $$R=\frac{TP}{TP+FN}$$

where true positive (TP) means that the predicted target is correct, false positive (FP) means that the predicted target type is wrong, and false negative (FN) means that the target was not predicted.

The mAP is a metric used to evaluate the recognition performance of a model across all categories. A higher mAP value indicates better model performance. mAP@0.5 refers to the average accuracy at a threshold value of 0.5. Similarly, mAP@0.75 refers to the average accuracy at a threshold value of 0.75. Finally, mAP@0.5:0.95 refers to the average accuracy over a range of threshold values from 0.5 to 0.95, with a step of 0.05. This value is calculated using the equation shown in Equations (10) and (11).

(10) $$mAP=\frac{1}{N}\sum_{1}^{N}AP$$

(11) $$AP=\int_{0}^{1}P\left(R\right)dR$$

where N represents the number of categories in the training set and AP represents the accuracy of a single category. The F1 score is a metric that evaluates the accuracy of a classification model by considering both its precision and recall. It is calculated as shown in Equation (12).

(12) $$F_{1}=2\cdot \frac{Precision\cdot Recall}{Precision+Recall}$$

Parameter represents the number of parameters in the model, reflecting the size of the model; GFLOPs represent the number of floating point operations, reflecting the amount of computation in the model; and inference represents the time required to detect the image, reflecting the detection rate of the model.

### 3.4. Experimental Results

The training results for different printing defect types are shown in Table 5. Our proposed model shows significant improvement in the detection performance of all types of defects: the model is particularly effective in the detection of “White line” and “Offset” defects, especially the map@50 of “Offset” defects is improved by 10, and white line defects are improved by 1.6, which indicates that the model performs excellently in the detection of narrow and long defects. Furthermore, there is an overall improvement in the detection of subtle defects, such as “Dirty” and “Mark”, which proves the advantages of our network in detecting small printing defects.

### 3.5. Ablation Experimental

We create comparison experimental groups for each of the modifications to evaluate their ability to detect network performance. Table 6 demonstrates that the modifications made to the baselines significantly impact accuracy and mAP performance metrics. Compared to YOLOv5, optimizing the loss function to WEIOU results in a 0.2–1.1 improvement in mAP. Additionally, improving the network with LSK-RepConv and C3-DCN results in a significant performance boost, with mAP increasing by 1.9–2.4 and 0.5–1.6, respectively. When these two modules are combined, the mAP improves significantly by 2.3. Despite a slight increase in the number of parameters, each improvement strategy has a significant impact on performance.

**Figure 8:**
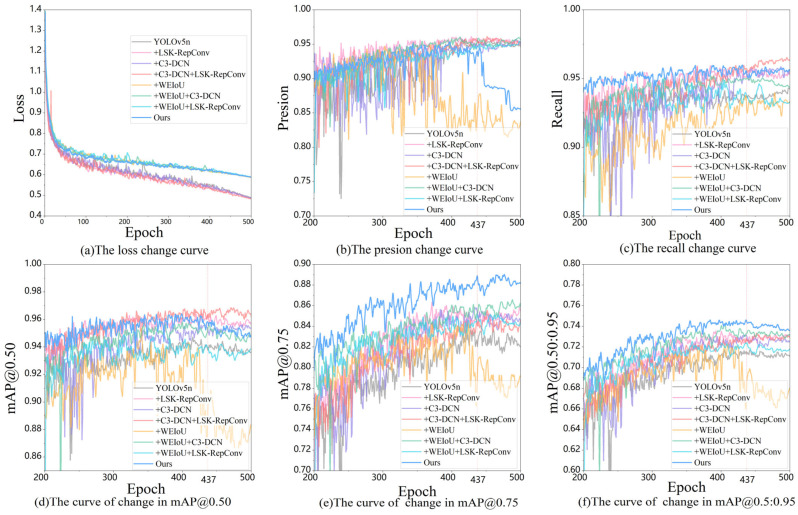
The performance changes in the model with different improvements.

Figure 8 shows training curves of total loss, precision, recall, mAP0.5, mAP0.75, and mAP0.5:0.95 during training, where ‘+LSK-RepConv’, ‘+C3-DCN’, ‘+WEioU’, and ‘Ours’ denote increased attention mechanism, deformable convolution, improved loss function, and our proposed model, respectively. Throughout training, the loss curve steadily decreases, while the accuracy, recall, and mAP curves at varying IoU thresholds (mAP@0.5, mAP@0.75, and mAP@0.5:0.95) continuously improve. Each improvement strategy outperforms the baseline YOLOv5 model individually, and the combined improvements result in optimal model performance, surpassing any single enhancement. (Ours and +WEiou achieved the highest performance in 437-epoch and 369-epoch training, respectively).

### 3.6. Comparison of Results of Pruning Algorithms

To evaluate model impact, we compare several common pruning methods. As shown in Table 7, the pruning operation effectively reduces the number of parameters in the model and the inference time. However, the LAMP and random methods have faster inference times, but they exhibit significantly poorer accuracy. Although slimming and group sparsity have comparable metrics to our adopted method, they do not perform as well as the Group Norm method in terms of model parameter count and occupancy. Compared to the L1 pruning method, the Group Norm method achieves higher accuracy and is better suited for real production environments where a low false detection rate is crucial. Therefore, we have chosen to adopt the Group Norm method to reduce the model’s parameter count and inference time.

**Figure 9:**
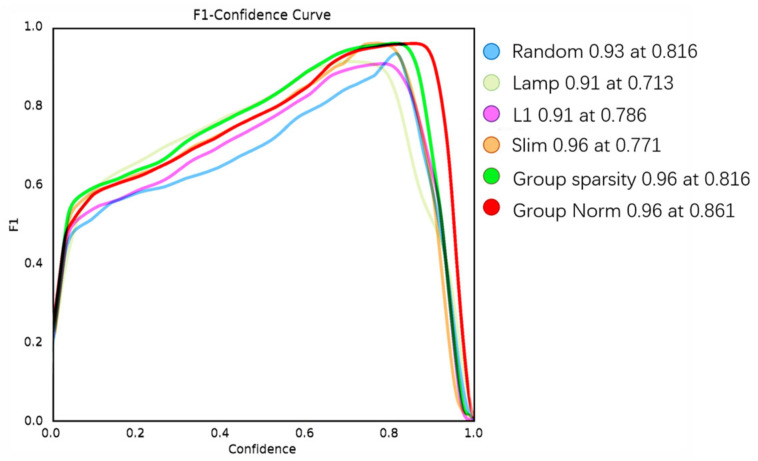
F1 scores achieved through different pruning techniques.

Figure 9 shows the F1 score after pruning for each method. It is evident that the Group Norm method yields a higher F1 score, achieving 0.96 at a confidence threshold of 0.861, which is superior to the other methods.

**Figure 10:**
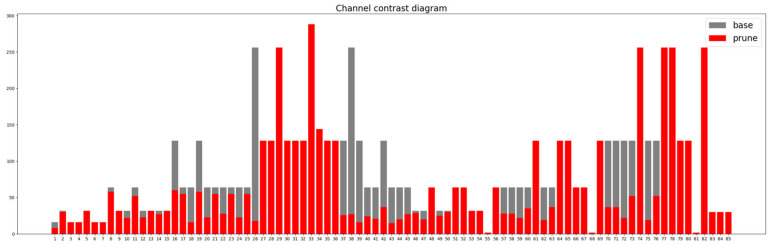
Comparison of the number of channels before and after Group Norm pruning method.

Figure 10 illustrates the change in the number of channels in each layer before and after applying the Group Norm pruning method to the model. After pruning, the model retains only 664,675 parameters, a 65.22% reduction, with an overall pruning rate of 71.69%. Although the recall of the pruned model slightly decreases by 0.3% and the mAP0.5:0.95 decreases by 0.4, the model demonstrates superior detection capabilities. Additionally, the inference time has decreased from 3.74 ms to 3.0 ms, and the frame rate has increased by 55.7%. These results demonstrate the effectiveness of the pruning algorithm in significantly reducing model complexity.

### 3.7. Comparative Experiments

To demonstrate the effectiveness and superiority of our proposed detection method, we trained and tested lightweight models, including YOLOv3, YOLOv7, Mobilenetv3, Shufflenetv2, etc., on the same dataset and compared their experimental performance with our proposed model.

Table 8 shows that our proposed model achieves the best performance in terms of mAP, with 95.7 and 74.1 on mAP@0.5 and mAP@0.5:0.95 metrics, respectively, compared to other lightweight networks, even with an 8% increase in the number of parameters compared to the baseline model, before the pruning operation. After model pruning and fine-tuning operations, mAP@0.5:0.95 intersection over union decreased by only 0.5, while the number of parameters is significantly reduced by 65%, resulting in a lightweight design with a 3.0 ms inference time and a 2.1 Mb model size. 

**Figure 11:**
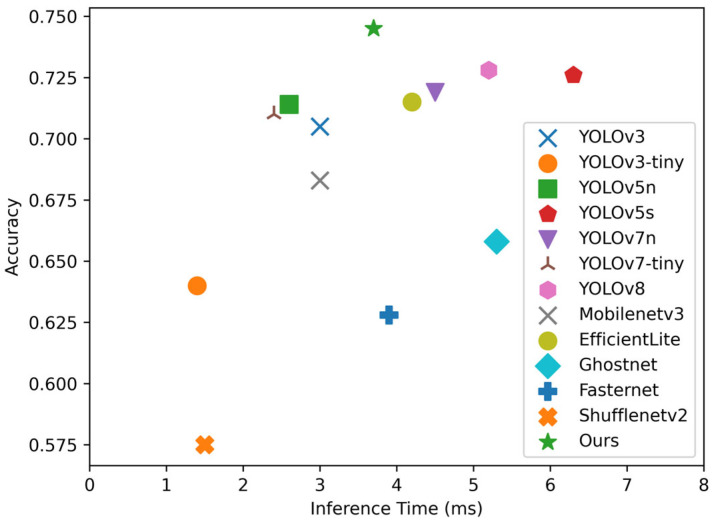
Inferencing time and mAP@0.5:0.95 for different lightweight networks.

A comparison of the detection performance of different lightweight models is shown in Figure 11. Although our proposed model is slower than YOLOv7-tiny in terms of inference time, it has only 43.8% of the parameters and an improved mAP of 3.1 compared to it, which demonstrates the suitability of our model for deployment on low-computing power devices.

**Figure 12:**
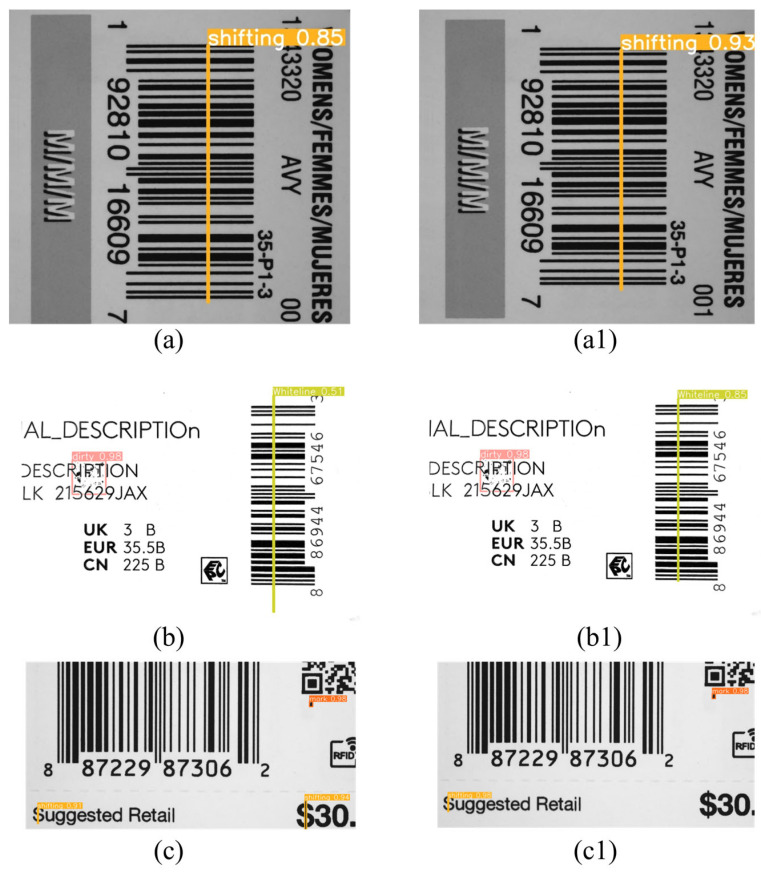
Detection result comparison ((**a**–**c**): YOLOv5 and (**a1**–**c1**): improved YOLOv5).

Figure 12 compares the results of the baseline model and our proposed model for detecting defects in variable data printing. The comparison shows that our model has a lower false detection rate and higher accuracy than the baseline model in detecting different background images with different defect types; this result shows that our improvement has advanced performance. Overall, the method proposed in this paper outperforms other lightweight algorithms and can identify printing defects quickly and accurately, providing better support for subsequent defect information analysis.

## 4. Conclusions

This paper introduces an enhanced printing defect detection model based on YOLOv5, addressing the shortcomings of existing models in detecting small defects and narrow and long-shaped defects, which are characteristic of printing defects. By combining improved C3-DCN and LSK-RepConv modules and adopting the WEIoU loss function to optimize the feature extraction performance of the model, the feature extraction performance is optimized and the ability to detect the special shape and small-size defects is enhanced. Finally, we adopt the Group Norm pruning method, which significantly reduces the inference time and parameter number of the model. The experimental results demonstrate a 2.7 enhancement in mAP@0.5 and a 20% reduction in Inference time in comparison to the baseline network. Compared with the commonly used lightweight target detection model, the proposed model can achieve 0.741 mAP50:95 and 0.66 M model parameters, the guaranteed accuracy can reach 2.2Gflops, and the inference time is shortened to 3.0 × 10^−3^ ms, which achieves the highest accuracy and is sufficient to meet the requirements of defective printing detection, detect defective products in time, and reduce the production cost of enterprises. The defect information obtained from the model lays a solid foundation for the subsequent defect assessment and analysis and the construction of traceability system. The future focus is to deploy the improved model into resource-constrained embedded devices, while exploring new defect samples and introducing new network structures to learn and recognize samples, with the objective of further improving the robustness of the model.

## Figures and Tables

**Table 1:** A comparative analysis of the YOLO algorithm series.

Model	Precision (%)	Recall (%)	mAP@0.5	mAP@0.5:0.95	Parameter	GFLOPs
YOLOv3	0.952	0.921	0.941	0.705	2,336,818	5.9
YOLOv3-tiny	0.949	0.900	0.928	0.640	547,700	1.0
YOLOv5s	0.938	0.922	0.936	0.726	7,023,610	15.8
YOLOv5n	0.951	0.938	0.939	0.714	1,765,930	4.1
YOLOv7	0.948	0.939	0.936	0.719	2,346,204	6.9
YOLOv7-tiny	0.945	0.930	0.925	0.710	1,517,444	3.4
YOLOv8n	0.950	0.941	0.942	0.728	3,011,807	8.2

**Table 2:** Comparison of results with different loss functions.

IoU Name	mAP@0.5	mAP@0.75	mAP@0.5:0.95
CIoU	0.939	0.838	0.714
GIoU	0.935	0.830	0.712
EIoU	0.943	0.842	0.72
DIoU	0.876	0.745	0.654
WEIoU	0.941	0.846	0.725

**Table 3:** Distribution of various defects in the dataset.

Dataset	Number of Images	Satellite Ink Spot	White Line	Mark	Offset	Dirty
Train	3000	630	580	800	670	950
Validation	360	80	74	105	65	127
Test	360	77	80	96	70	120
Total	3720	787	734	1001	805	1197

**Table 4:** Experimental setting.

Parameter	Value
Init_lr	0.02
Batchsize	8
Lr decay	0.01 (0.02 × 0.01)
Epoch	500
Box loss gain	0.5
Momentum	0.937
Optimizer	Adam
Learning rate schedule	Linear

**Table 5:** The results of different types of defects.

Defect Class	Precision (%)	Recall (%)	mAP@0.5	mAP@0.75	mAP@0.5:0.95
Satellite ink spot	0.991	0.996	0.995→0.995	0.973→0.995	0.874→0.892
White line	0.961	0.943	0.957→0.973	0.853→0.894	0.741→0.751
Mark	0.987	0.980	0.984→0.985	0.925→0.950	0.726→0.740
Offset	0.849	0.861	0.767→0.868	0.519→0.648	0.469→0.547
Dirty	0.983	0.986	0.985→0.985	0.920→0.944	0.760→0.777

**Table 6:** The results of the model with different improvements.

Method	Precision (%)	Recall (%)	mAP@0.5	mAP@0.75	mAP@0.5:0.95	Params
Baseline (YOLOv5n)	0.951	0.938	0.939	0.838	0.714	1.76
+LSK-Rep	0.950	0.954	0.960	0.862	0.733	1.95
+C3-DCN	0.955	0.944	0.955	0.851	0.719	1.74
+LSK-Rep+C3-DCN	0.960	0.959	0.966	0.855	0.732	1.91
+WEiou	0.956	0.934	0.941	0.846	0.725	1.76
+WEiou+C3-DCN	0.947	0.937	0.941	0.851	0.724	1.72
+WEiou+LSK-Rep	0.956	0.946	0.946	0.867	0.733	1.95
Ours	0.941	0.956	0.957	0.890	0.745	1.91

**Table 7:** The results of the model with different pruning methods.

Name	Precision (%)	Recall (%)	mAP@0.5	mAP@0.75	mAP@0.5:0.95	Parameters	FPS
Before pruning	0.941	0.956	0.957	0.890	0.745	1,910,892	267.5
LAMP [24]	0.934	0.901	0.934 (−0.023)	0.782 (−0.108)	0.676 (−0.069)	539,915 (28.25%)	339.1
Random pruning	0.918	0.939	0.950 (−0.007)	0.847 (−0.043)	0.719 (−0.026)	1,039,981 (54.42%)	332.9
L1 [25]	0.939	0.958	0.966 (+0.009)	0.886 (−0.004)	0.745 (0.000)	650,468 (34.04%)	325.5
Slimming [26]	0.966	0.952	0.958 (+0.001)	0.888 (−0.002)	0.744 (−0.001)	863,491 (45.19%)	328.2
Group sparsity [23]	0.953	0.956	0.964 (+0.007)	0.878 (−0.012)	0.742 (−0.003)	776,110 (40.62%)	335.9
Group Norm [23]	0.954	0.953	0.961 (+0.004)	0.886 (−0.004)	0.741 (−0.004)	664,675 (34.78%)	323.2

**Table 8:** The training results of different models.

Model	mAP@0.5	Map@0.5:0.95	Parameters	GFLOPs	Inference/ms (bs = 16)
YOLOv3	0.941	0.705	2,336,818	5.9	3.0 × 10^−3^
YOLOv3-tiny	0.928	0.64	547,700	1.0	1.4 × 10^−3^
YOLOv5s	0.936	0.726	7,023,610	15.8	6.3 × 10^−3^
YOLOv5n	0.939	0.714	1,765,930	4.1	2.6 × 10^−3^
YOLOv7 [27]	0.936	0.719	2,346,204	6.9	4.5 × 10^−3^
YOLOv7-tiny [27]	0.925	0.710	1,517,444	3.4	2.4 × 10^−3^
YOLOv8 [28]	0.942	0.728	3,011,807	8.2	5.2 × 10^−3^
Mobilenetv3 [29]	0.948	0.683	1,337,884	2.2	3.0 × 10^−3^
Shufflenetv2 [30]	0.829	0.575	813,254	1.5	1.5 × 10^−3^
Fasternet [31]	0.836	0.628	3,191,646	7.2	3.9 × 10^−3^
Ghostnet [32]	0.866	0.658	2,531,106	3.3	5.3 × 10^−3^
EfficientLite [33]	0.948	0.715	1,005,214	2.2	4.2 × 10^−3^
Improved-YOLOv5 (before pruning)	0.957	0.745	1,910,892	4.5	3.7 × 10^−3^
Improved-YOLOv5 (pruning)	0.961	0.741	664,675	2.2	3.0 × 10^−3^

## Data Availability

Under reasonable requirements, the dataset used in this study can be obtained from the corresponding authors.

## References

[1] Shi Y., Hou B., Liu J., Liu A., Guo S., Liu J. Element Defective Sample Augmentation Method Based on Improved DCGAN. Proceedings of the 2023 IEEE 16th International Conference on Electronic Measurement & Instruments (ICEMI).

[2] Valente A.C., Wada C., Neves D., Neves D., Perez F.V.M., Megeto G.A.S., Cascone M.H., Gomes O., Lin Q. Print defect mapping with semantic segmentation. Proceedings of the IEEE/CVF Winter Conference on Applications of Computer Vision.

[3] Ma B., Wei Z., Wang Y., Wu H. The defect detection of personalized print based on template matching. Proceedings of the 2017 IEEE International Conference on Unmanned Systems (ICUS).

[4] Son J.H., Kim C.O. (2021). A Study on the Application of Deep Learning Models for Real-Time Defect Detection in the Manufacturing Process: Cases of Defect detection in the Label Printing Process. J. Korea TAPPI.

[5] Betti A., Tucci M. (2023). YOLO-S: A lightweight and accurate YOLO-like network for small target detection in aerial imagery. Sensors.

[6] Luo J., Wang Q., Zou R., Wang Y., Liu F., Zheng H., Du S., Yuan C. (2023). A Heart Image Segmentation Method Based on Position Attention Mechanism and Inverted Pyramid. Sensors.

[7] Jing B., Duan P., Chen L., Du Y. (2023). EM-YOLO: An X-Ray Prohibited-Item-Detection Method Based on Edge and Material Information Fusion. Sensors.

[8] He K., Zhang X., Ren S., Sun J. (2015). Spatial pyramid pooling in deep convolutional networks for visual recognition. IEEE Trans. Pattern Anal. Mach. Intell..

[9] Ren S., He K., Girshick R., Sun J. (2016). Faster R-CNN: Towards real-time object detection with region proposal networks. IEEE Trans. Pattern Anal. Mach. Intell..

[10] Liu W., Anguelov D., Erhan D., Szegedy C., Reed S., Fu C.Y., Berg A.C., Leibe B., Matas J., Sebe N., Welling M. (2016). Ssd: Single shot multibox detector. European Conference on Computer Vision 2016.

[11] Bochkovskiy A., Wang C.-Y., Liao H.-Y.M. (2004). Yolov4: Optimal speed and accuracy of object detection. arXiv.

[12] Li J., Bai X., Pan J., Tian Q. A Deep Learning Method for Printing Defect Detection. Proceedings of the 2022 IEEE 4th International Conference on Power, Intelligent Computing and Systems (ICPICS).

[13] Liu A., Yang E., Wu J., Teng Y., Yu L. (2022). Double sparse low rank decomposition for irregular printed fabric defect detection. Neurocomputing.

[14] Zhang E., Ma Q., Chen Y., Duan J., Shao L. (2022). EGD-Net: Edge-Guided and differential attention network for surface defect detection. J. Ind. Inf. Integr..

[15] Li D., Li Y., Li J., Lu G., Wang L., Gall J., Chin T.-J., Sato I., Chellappa R. (2022). PPR-Net: Patch-Based multi-scale pyramid registration network for defect detection of printed labels. Asian Conference on Computer Vision 2022.

[16] Tang H., Shan L., Dan Y., Qiao Y. (2023). A visual defect detection for optics lens based on the YOLOv5-C3CA-SPPF network model. Opt. Express.

[17] Wang C.-Y., Liao H.-Y.M., Wu Y.-H., Chen P.-Y., Hsieh J.-W., Yeh I.-H. CSPNet: A new backbone that can enhance learning capability of CNN. Proceedings of the IEEE/CVF Conference on Computer Vision and Pattern Recognition Workshops.

[18] Dai J., Qi H., Xiong Y., Li Y., Zhang G., Hu H., Wei Y. Deformable convolutional networks. Proceedings of the IEEE International Conference on Computer Vision.

[19] Wang W., Dai J., Chen Z., Huang Z., Li Z., Zhu X., Hu X., Lu T., Lu L., Li H. Internimage: Exploring large-scale vision foundation models with deformable convolutions. Proceedings of the IEEE/CVF Conference on Computer Vision and Pattern Recognition.

[20] Li Y., Hou Q., Zheng Z., Cheng M.-M., Yang J., Li X. (2023). Large Selective Kernel Network for Remote Sensing Object Detection. arXiv.

[21] Zhang Y.-F., Ren W., Zhang Z., Jia Z., Wang L., Tan T. (2021). Focal and efficient IOU loss for accurate bounding box regression. arXiv.

[22] Wang J., Xu C., Yang W., Yu L. (2022). A normalized Gaussian Wasserstein distance for tiny object detection. arXiv.

[23] Fang G., Ma X., Song M., Mi M.B., Wang X. Depgraph: Towards any structural pruning. Proceedings of the IEEE/CVF Conference on Computer Vision and Pattern Recognition.

[24] Lee J., Park S., Mo S., Ahn S., Shin J. (2021). Layer-adaptive sparsity for the magnitude-based pruning. arXiv.

[25] Liu Z., Li J., Shen Z., Huang G., Yan S., Zhang C. Learning efficient convolutional networks through network slimming. Proceedings of the IEEE International Conference on Computer Vision.

[26] Hao L., Kadav A., Durdanovic I., Samet H., Graf H.P. (2017). Pruning filters for efficient convnets. arXiv.

[27] Wang C.-Y., Bochkovskiy A., Liao H.-Y.M. YOLOv7: Trainable bag-of-freebies sets new state-of-the-art for real-time object detectors. Proceedings of the IEEE/CVF Conference on Computer Vision and Pattern Recognition.

[28] Terven J., Córdova-Esparza D.-M., Romero-González J.-A. (2023). A comprehensive review of yolo architectures in computer vision: From yolov1 to yolov8 and yolo-nas. Mach. Learn. Knowl. Extr..

[29] Howard A., Mark S., Grace C., Chen L.-C., Chen B., Tan M., Wang W., Zhu Y., Pang P., Vasudevan V. Searching for mobilenetv3. Proceedings of the IEEE/CVF International Conference on Computer Vision.

[30] Ma N., Zhang X., Zheng H.-T., Sun J. Shufflenet v2: Practical guidelines for efficient cnn architecture design. Proceedings of the European Conference on Computer Vision (ECCV).

[31] Chen J., Kao S., He H., Zhuo W., Wen S., Lee C.-H., Chan G.S.-H. Run, Don’t walk: Chasing higher FLOPS for faster neural networks. Proceedings of the IEEE/CVF Conference on Computer Vision and Pattern Recognition.

[32] Han K., Wang Y., Tian Q., Guo J., Xu C., Xu C. Ghostnet: More features from cheap operations. Proceedings of the IEEE/CVF Conference on Computer Vision and Pattern Recognition.

[33] Tan M., Le Q.V. (2019). Efficientnet: Rethinking model scaling for convolutional neural networks. arXiv.

